# Imaging approach to retroperitoneal vascular tumors: a narrative review

**DOI:** 10.1007/s00261-025-05332-w

**Published:** 2025-12-11

**Authors:** Arleen Li, Ellen L. Wolf, Shabnam Fidvi

**Affiliations:** Department of Radiology, Montefiore Medical Center, Bronx, USA

**Keywords:** Retroperitoneal vascular tumors, Imaging features

## Abstract

Primary retroperitoneal tumors are rare and can encompass a large range of pathologies, including both benign and malignant etiologies. Retroperitoneal tumors with a vascular component constitute a small percentage of these tumors, and include intravascular and extravascular leiomyosarcoma, paraganglioma, extrarenal angiomyolipoma, Castleman disease, angiosarcoma, hemangioma, cystic lymphangioma, and inflammatory myofibroblastic tumor. Cross-sectional imaging is often valuable to characterize vascular retroperitoneal masses and delineate their relationship with surrounding anatomical structures, aiding in surgical planning. This article aims to review the imaging features of these vascular retroperitoneal tumors and presents a diagnostic approach to aid in diagnosing these tumors.

## Introduction

Retroperitoneal tumors encompass a large range of pathologies, owing partially to the large size of the retroperitoneum and the multiple organs and cell types that can be found within it. [1] Primary retroperitoneal tumors are rare, comprising approximately 0.1-0.2% of all malignancies. [1] Retroperitoneal tumors with a vascular component constitute only a small subset of these tumors. In this article, we aim to review the imaging features of vascular retroperitoneal tumors and masses, including intravascular and extravascular leiomyosarcoma, paraganglioma, extrarenal angiomyolipoma, Castleman disease, angiosarcoma, hemangioma, cystic lymphangioma, and inflammatory myofibroblastic tumor. Although there is an overlap of imaging findings in these tumors, a proposed approach to making a definitive diagnosis based on the imaging findings is presented in Table 1.

**Table 1:**
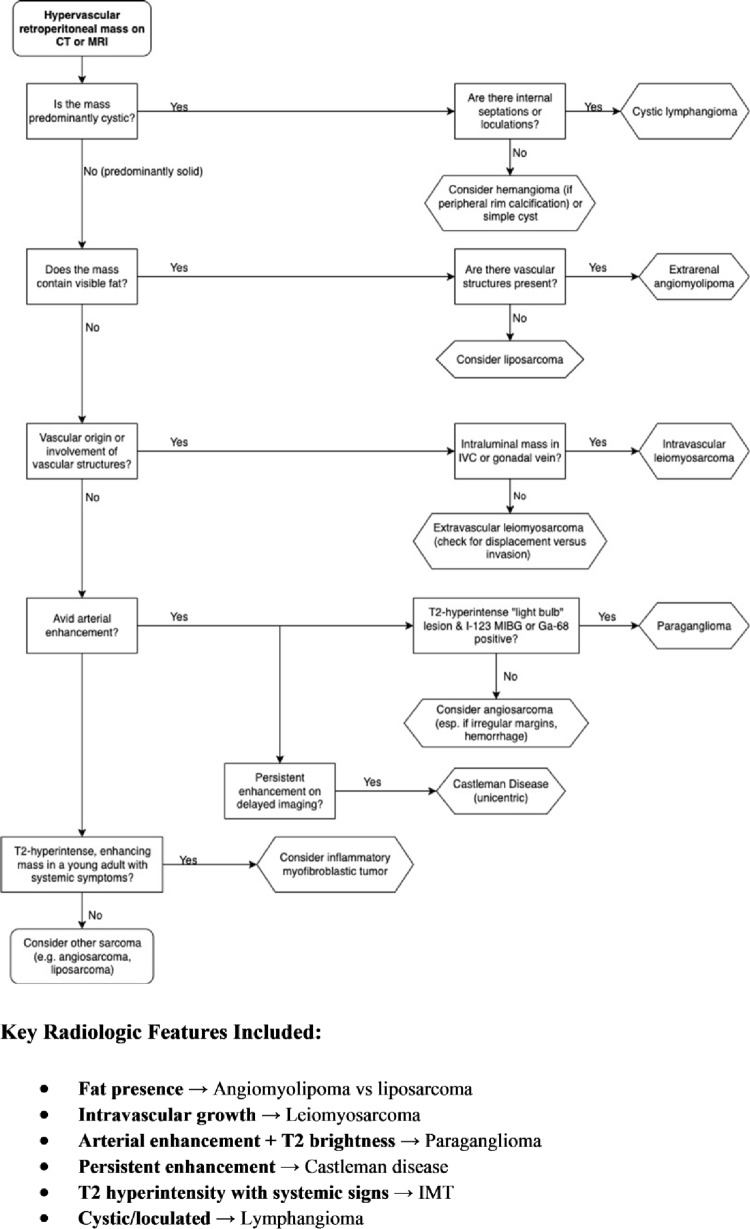
Proposed diagnostic algorithm for hypervascular retroperitoneal masses on CT or MRI. Created with assistance from ChatGPT (OpenAI. ChatGPT (June 2024 version) [Large Language model]. https://chat.openai.com)

## Methods

Literature review was performed via database search on PubMed and Web of Science using “retroperitoneal” and the diagnostic entities (e.g. “leiomyosarcoma”) as keywords. Publications pertaining to vascular retroperitoneal lesions in children were excluded. Articles with a focus on radiologic diagnostic approach and specific imaging findings were preferentially selected. No specific limit on time of publication was implemented.

## Leiomyosarcomas

**Fig. 1:**
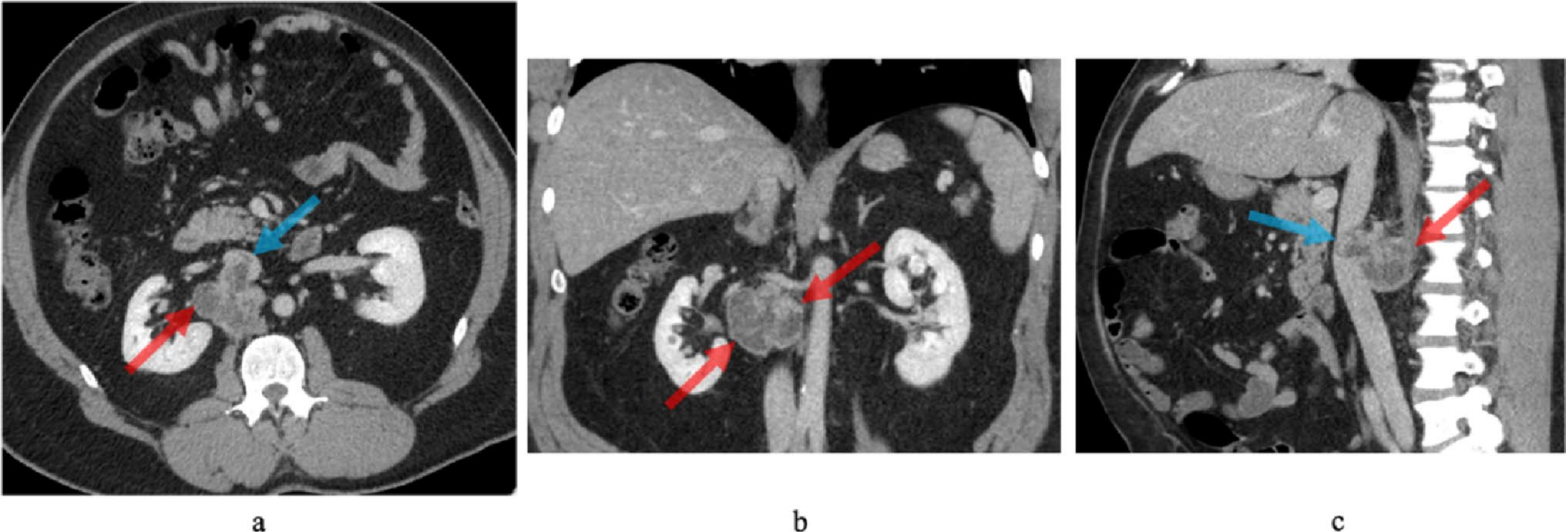
Axial (**a**), coronal (**b**) and sagittal (**c**) contrast-enhanced CT images through the mid-abdomen show a heterogeneously enhancing right perirenal retroperitoneal mass with a large extravascular component (red arrows) and a small intravascular IVC component (blue arrows). Pathology confirmed leiomyosarcoma arising from the IVC

**Fig. 2:**
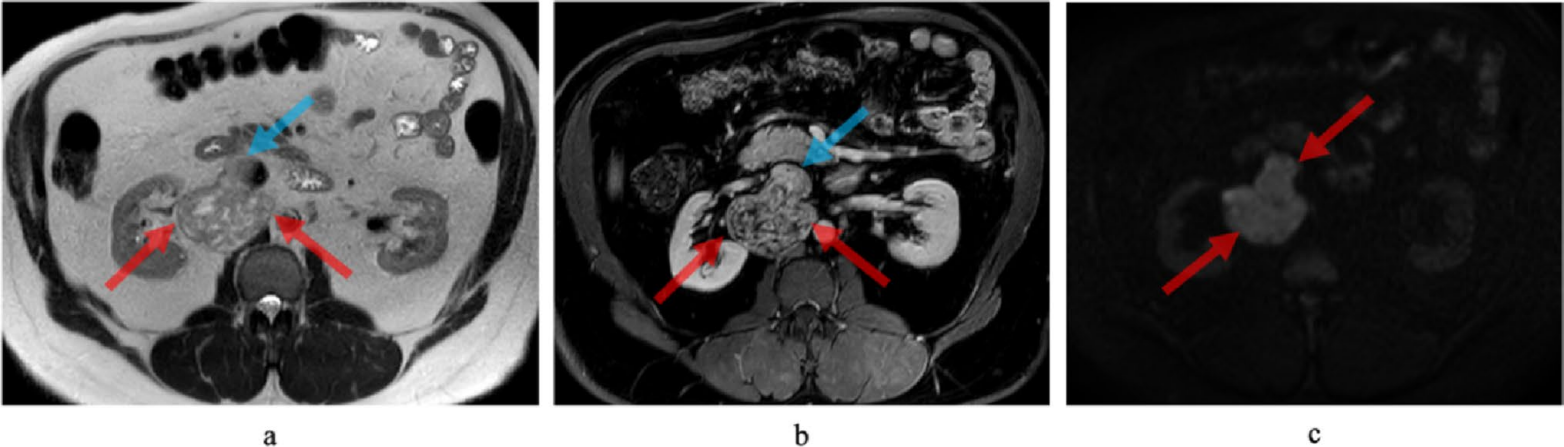
Axial T2-weighted image (**a**) without fat saturation and axial fat-saturated T1-weighted contrast enhanced image (**b**) demonstrate a heterogeneously enhancing right perirenal retroperitoneal mass with a large extravascular component (red arrows) and a small intravascular IVC component (blue arrows). Diffusion weighted image (**c**) shows high restriction diffusion within the mass (red arrows). Pathology confirmed leiomyosarcoma arising from the IVC

Leiomyosarcomas are the second most common primary retroperitoneal tumor and the second most common sarcoma in adults. [2–3] They are the most common sarcomas arising from large retroperitoneal blood vessels, especially the inferior vena cava (IVC). The majority of cases are completely extravascular (or extraluminal), although retroperitoneal leiomyosarcomas can also present with both extravascular and intravascular components (Figs. 1 and 2), and rarely as completely intravascular (or intraluminal) masses. [2–3] Leiomyosarcomas arise from smooth muscle tissue within the retroperitoneum, from retroperitoneal venous walls, or from embryonic Wolffian remnants. [2] These tumors are most frequently diagnosed in patients between the ages of 54 and 65 years and occur more often in women than in men. [3]

Retroperitoneal leiomyosarcomas are typically asymptomatic when small due to their relative sparing of adjacent visceral structures and are usually therefore detected incidentally. Symptoms may present when the tumor compresses adjacent organs, resulting in pain or they may present with nonspecific symptoms, such as malaise, weight loss, and nausea or vomiting. [3–4] Leiomyosarcomas with intravascular components tend to be symptomatic earlier. Specific symptoms depend on the tumor’s location. Involvement of the suprahepatic IVC may obstruct the hepatic veins and result in Budd-Chiari syndrome. On the other hand, involvement of the mid-IVC and/or renal veins may lead to renal dysfunction and/or right upper quadrant pain. [4] Involvement of the infrarenal IVC can result in lower extremity edema. [3]

### Extravascular leiomyosarcoma

**Fig. 3:**
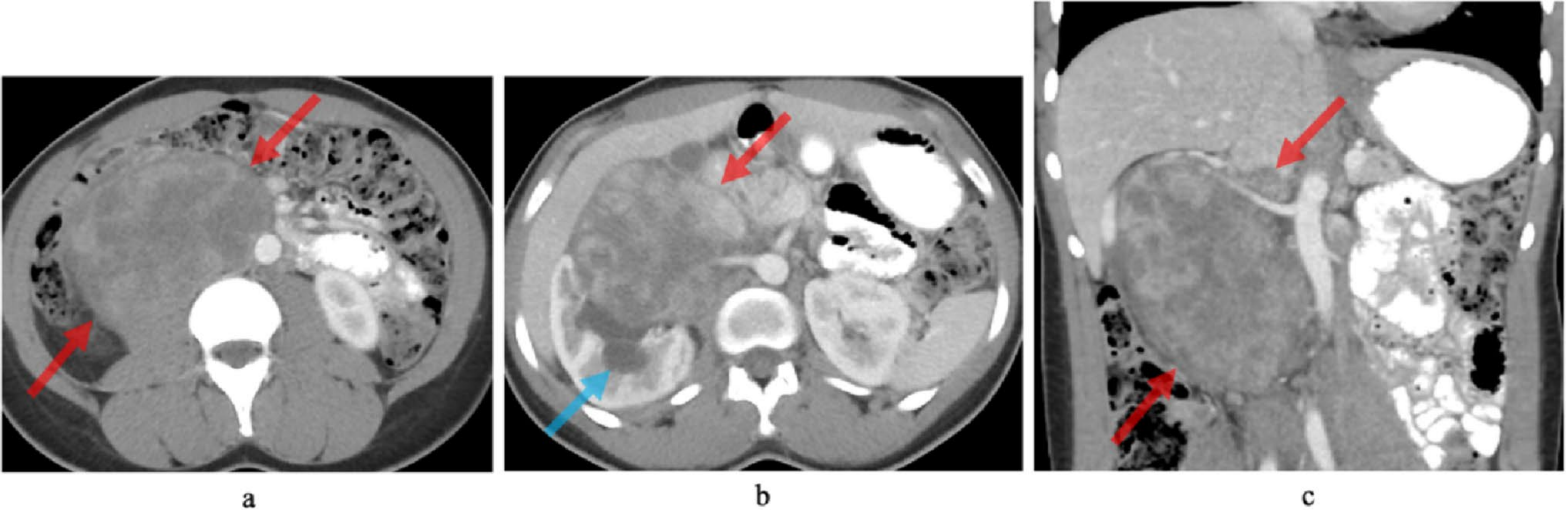
Axial (**a**, **b**) and coronal (**c**) contrast-enhanced CT images show a large heterogeneously enhancing right retroperitoneal mass (red arrows). The lesion has low density components and exerts mass effect on the right kidney with hydronephrosis (blue arrow). The patient had biopsy proven leiomyosarcoma arising from the IVC

The majority of retroperitoneal leiomyosarcomas are extravascular. These tumors initially displace adjacent organs, as they tend to expand along tissue planes of least resistance, allowing them to grow to potentially large sizes before detection. [2] Tumors can often grow to greater than 10 cm. [2] On computed tomography (CT) imaging, retroperitoneal leiomyosarcomas typically appear as large soft tissue masses and often demonstrate areas of necrosis, hemorrhage, and/or cystic change (Figure 3). Calcifications are uncommon. [2–3] These tumors almost always enhance heterogeneously, with hyperenhancement of the solid components of the tumor relative to muscle in 56-61% of cases. [3] Collateral vessels are also often seen. Although leiomyosarcomas tend to initially displace adjacent organs, subsequent invasion of adjacent structures is common. These may include the kidney, liver, adrenal gland, pancreas, stomach, and spine. [3] Hematogenous metastases to the lungs and liver are more common compared to lymphatic spread. [3]

On ultrasound, these tumors usually appear as solid masses with lobulations. They often demonstrate irregular cystic spaces, which may be anechoic or may contain hypoechoic material, secondary to necrosis and/or hemorrhage. [3]

**Fig. 4:**
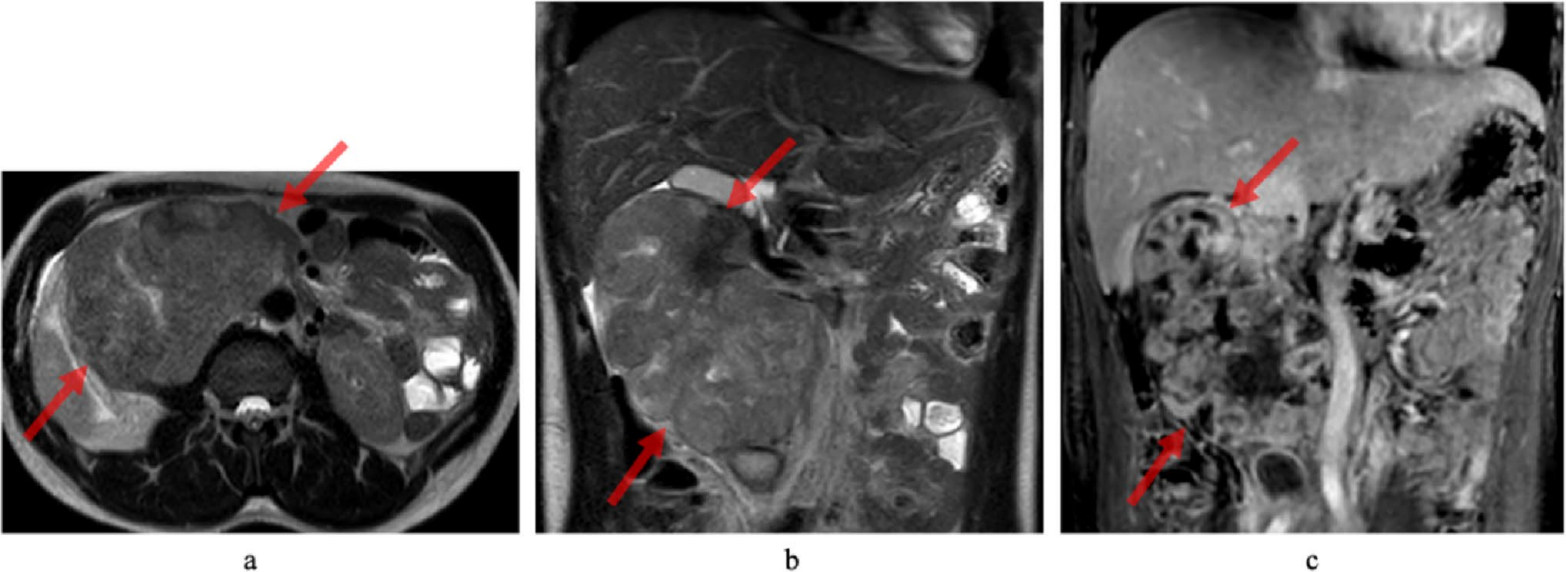
Axial and coronal T2-weighted images (**a**, **b**) without fat saturation and a coronal fat-saturated T1-weighted contrast-enhanced image (**c**) demonstrate a large heterogeneously enhancing right retroperitoneal mass (red arrows). The patient had biopsy proven leiomyosarcoma arising from the IVC

On magnetic resonance imaging (MRI), the solid components of the tumor typically demonstrate hypointensity to iso-intensity on T1-weighted images and hyperintensity on T2-weighted images. Areas of necrosis demonstrate T1 hypointensity and T2 hyperintensity compared to muscle. Hemorrhagic areas may demonstrate T1 hyperintensity. Contrast-enhanced images typically demonstrate heterogeneous enhancement, similar to CT (Figure 4). [3]

### Primary intravascular leiomyosarcoma

Retroperitoneal leiomyosarcomas growing in an entirely intravascular or intraluminal pattern are rare. The most common vessel involved in the retroperitoneum is the IVC, with the most common segment being the portion between the hepatic veins and renal veins. [3] Leiomyosarcomas with intravascular components are more commonly metastatic at the time of diagnosis compared to extravascular leiomyosarcomas. [3]

**Fig. 5:**
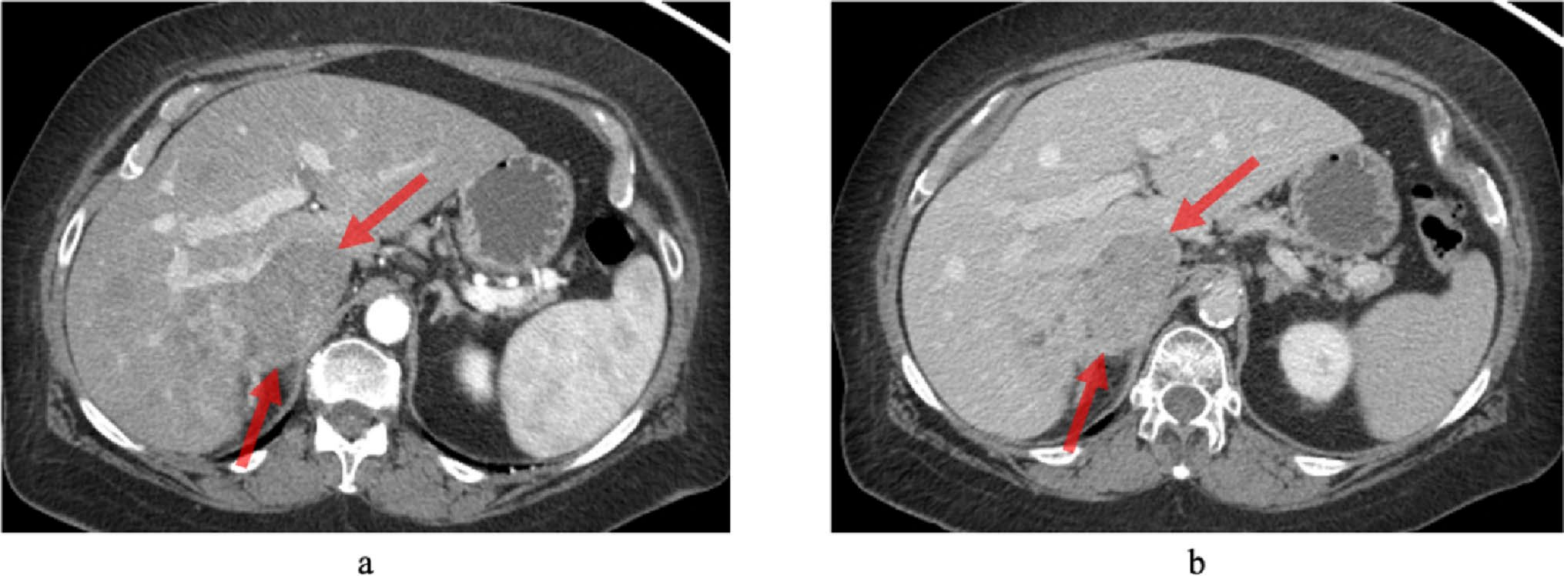
Intravascular Leiomyosarcoma. Axial contrast-enhanced CT images through the upper abdomen in arterial (**a**) and portal venous (**b**) phases demonstrate a large heterogeneously enhancing mass expanding and obliterating the lumen of the suprarenal IVC (red arrows). This is the imperceptible IVC sign, indicating an intravascular tumor

**Fig. 6:**
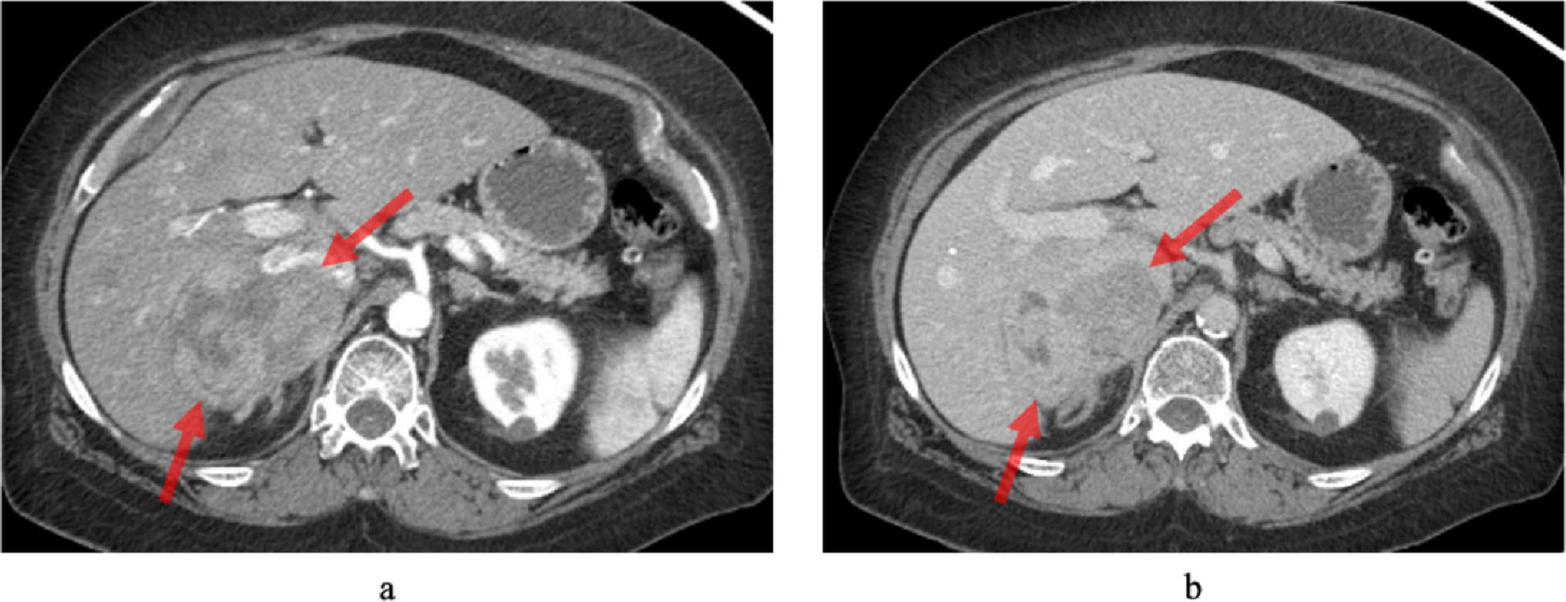
Axial contrast-enhanced CT images one level below that of Fig. 5 in arterial (**a**) and portal venous phases (**b**) demonstrate a large contiguous extravascular tumor component with similar enhancement characteristics (red arrows)

**Fig. 7:**
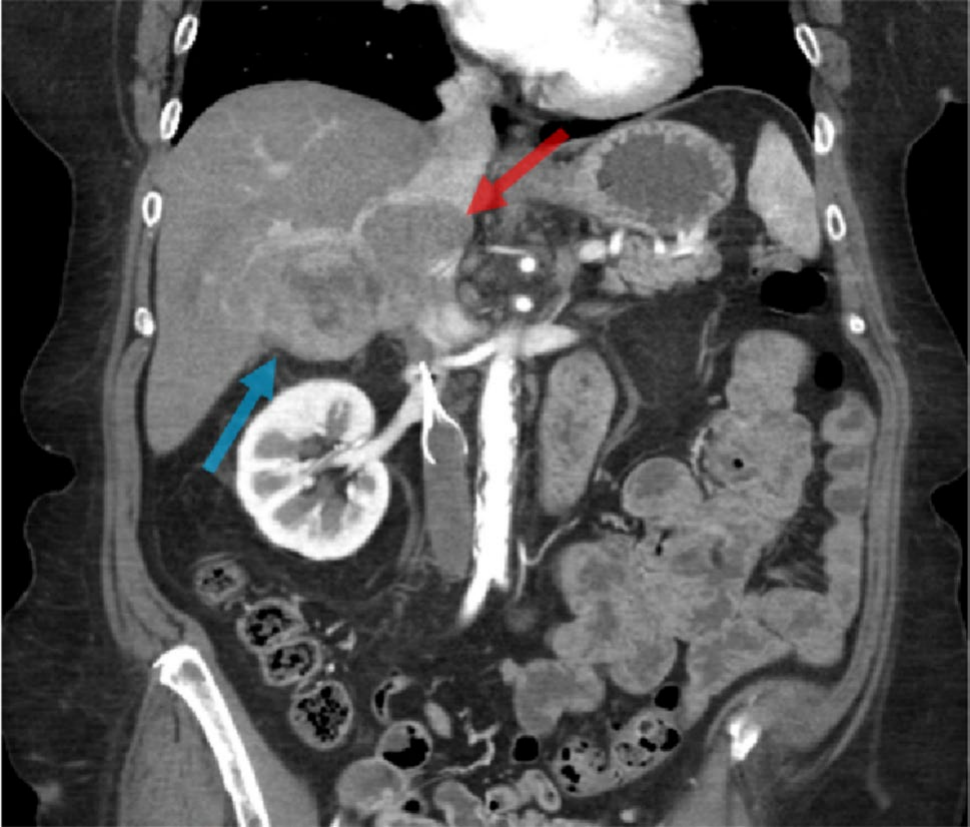
Coronal contrast-enhanced CT image in the arterial phase demonstrates a large bilobed heterogeneously enhancing suprarenal IVC mass with both intravascular (red arrow) and extravascular components (blue arrow). The patient has a IVC filter. Pathology confirmed leiomyosarcoma arising from the IVC

The imaging findings are similar to those of other tumor thrombi, including heterogeneous enhancement of the intraluminal tumor and dilatation of the IVC, if involved. [3] Obstruction of the vessel lumen may occur, with resultant development of extensive collateral vasculature. One useful finding described for intravascular leiomyosarcoma is an imperceptible IVC at the level of its contact with the mass. [4–5] (Figs. 5, 6, and 7). A tumor of extravascular origin would tend to compress the IVC instead. [4]

MRI may be helpful to further demonstrate the degree of tumor extension and differentiate tumor from bland thrombus. Black-blood sequences in particular delineate the degree of intraluminal tumor extension well, given the contrast between the hypointense intravascular blood and the hyperintense tumor. [3] Gradient-echo images may alternatively demonstrate hypointense tumor relative to high intraluminal signal within the vessels. [2] An enlarged IVC, increased T2 signal within the mass, and internal enhancement are more suggestive of IVC leiomyosarcoma as opposed to bland thrombus. [3–4] MRI may also help detect extensive collateral vessels which may develop due to slow growth of the tumor. [4] The tumor typically demonstrates similar signal characteristics as extravascular leiomyosarcoma: hypointense on T1-weighted sequences and hyperintense on T2-weighted sequences. [4] Metastases often occur via hematogenous spread and tumor may also subsequently metastasize lymphatically. Common sites of metastases include the liver, lungs, and lymph nodes. Metastatic lesions usually demonstrate similar MRI signal characteristics as the primary tumor. [4]

Ultrasound imaging typically shows an intraluminal solid mass with internal Doppler flow. [3] Large tumors tend to be heterogeneous, while smaller tumors demonstrate homogeneity. [3] Abnormal or even absent Doppler flow within the involved vessel may be seen. [4] 18 F-fluorodeoxyglucose positron emission tomography (FDG PET) can potentially be helpful to evaluate the extent of disease, although its true role in diagnosis and management of IVC leiomyosarcoma has not been established. [4]

**Fig. 8:**
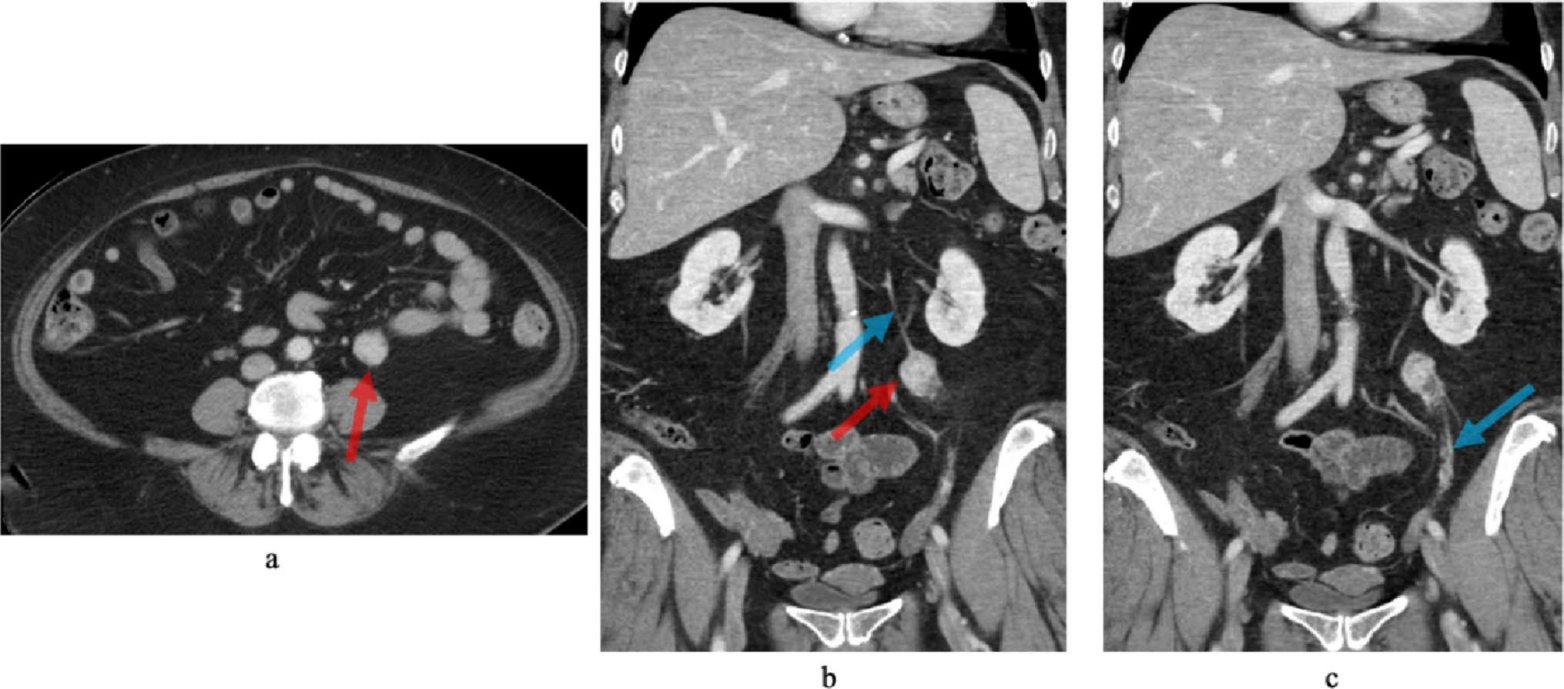
Leiomyosarcoma of the left gonadal vein. Axial (**a**) and coronal (**b**, **c**) contrast-enhanced CT images show an ovoid heterogeneously enhancing left retroperitoneal mass (red arrows) along the course of the left ovarian vein (blue arrows)

**Fig. 9:**
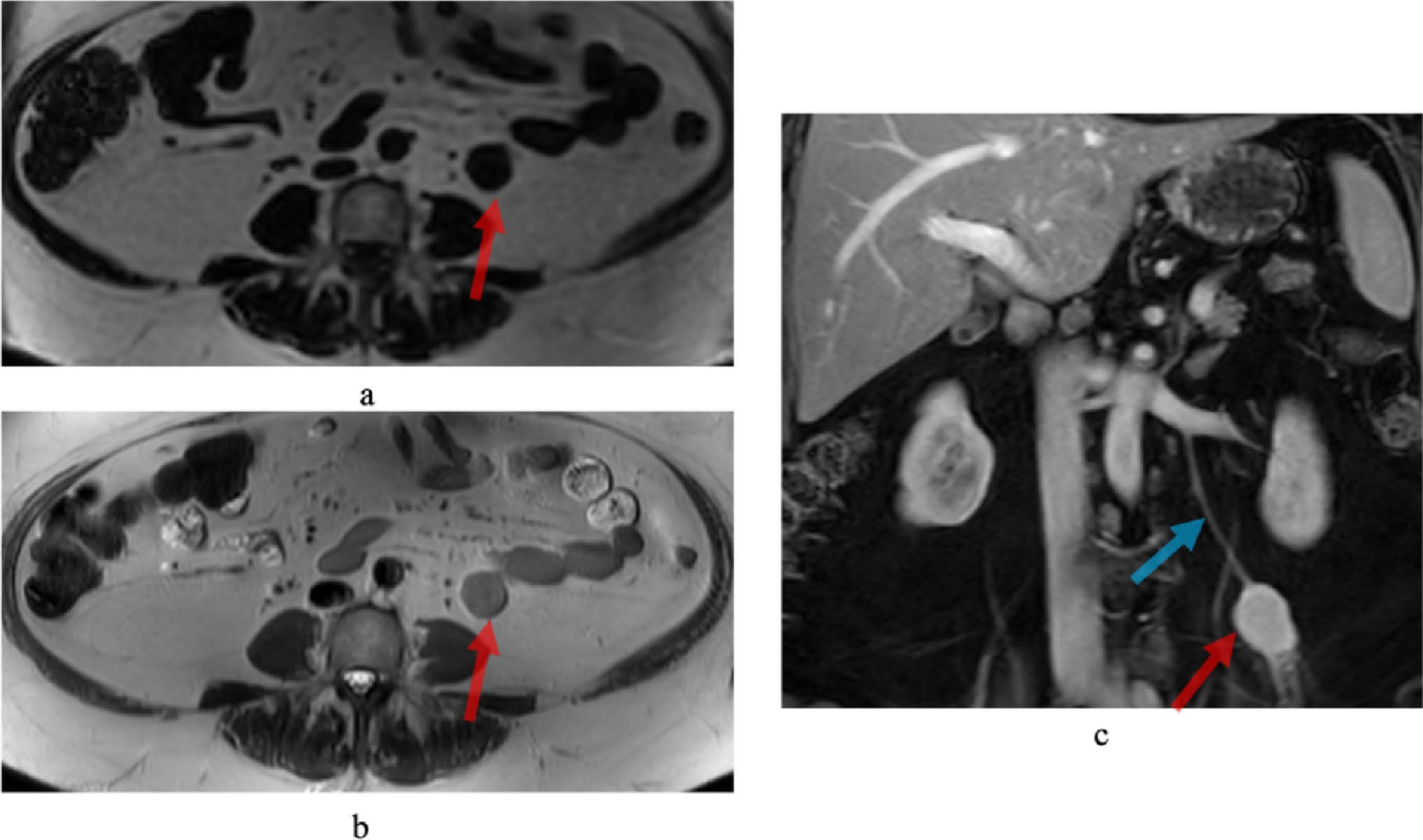
Axial T1 and T2-weighted images (**a**, **b**) without fat saturation demonstrate the mass to be T1 hypointense and moderately T2 hyperintense (red arrows). A coronal fat-saturated T1-weighted contrast enhanced image (**c**) demonstrates the mass (red arrow) to be avidly enhancing and contiguous with the left ovarian vein (blue arrow)

Leiomyosarcomas involving the gonadal veins have been reported in the literature, but are extremely rare. [6] Gonadal vein leiomyosarcoma most commonly presents as a large heterogeneous enhancing mass replacing the normal gonadal vein and often in a longitudinal orientation. [7] (Figs. 8 and 9). As with IVC leiomyosarcomas, cystic, necrotic, and hemorrhagic areas may be seen. [7]

## Paragangliomas

Paragangliomas are tumors arising from chromaffin cells, which may originate from the adrenal glands or may be extra-adrenal in location. Paragangliomas arising from the adrenal glands are referred to as pheochromocytomas. [1, 3] These tumors are most commonly encountered in adults aged 20-40 years. [3] They may be associated with several clinical syndromes, including Von Hippel-Lindau syndrome, Multiple Endocrine Neoplasia syndrome Types 2 A and 2B, Neurofibromatosis type 1, and Carney triad. [3] Some paragangliomas may present with clinical findings of catecholamine excess, such as hypertension and elevated urinary metanephrines. Rarely, paragangliomas may first come to attention with retroperitoneal hemorrhage resulting in an acute abdomen. [1] Extra-adrenal paragangliomas occur most commonly at the organ of Zuckerkandl, although they can occur anywhere in the retroperitoneum. [3]

Typical imaging features of paragangliomas on CT include large lobulated well-marginated soft tissue masses with intense early enhancement due to their vascularity. Necrosis and hemorrhage are common with larger lesions. Calcifications are seen in 15% of cases. Although some imaging findings overlap with leiomyosarcoma, avid enhancement, calcifications, and location at the organ of Zuckerkandl are more suggestive of paraganglioma. [3] 22-50% of extra-adrenal paragangliomas are malignant. Malignant tumors are more likely to have irregular margins and more extensive necrosis. [1] On MRI, they are heterogeneously hyperintense on T2-weighted sequences. They are typically T1-hypointense, although areas of T1 hyperintensity may be seen in regions of hemorrhage. Fluid-fluid or fluid-hemorrhage levels may also be seen. [1, 3]

**Fig. 10:**
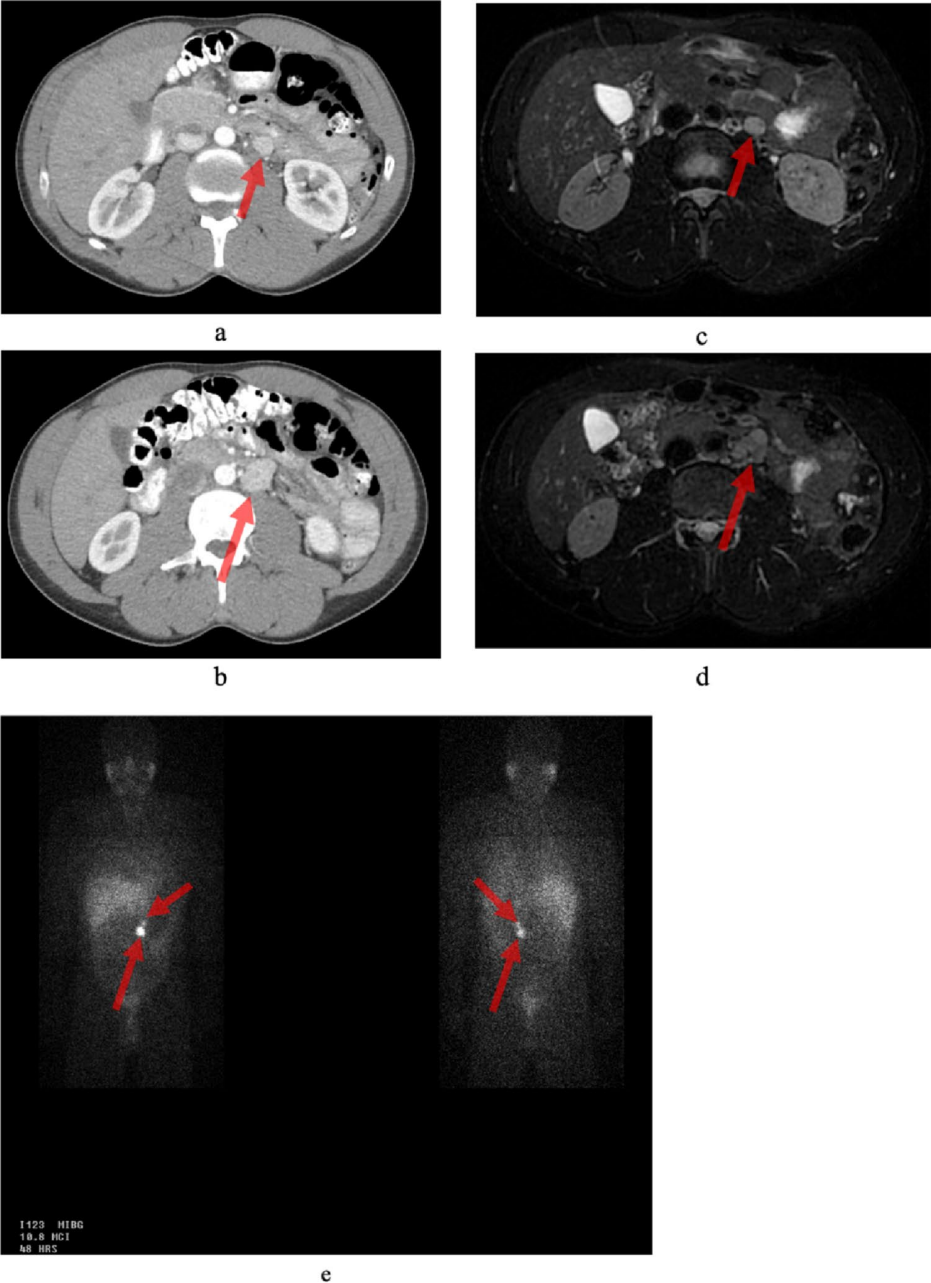
Two left retroperitoneal paragangliomas are demonstrated on CT, MRI, and I-123 metaiodobenzylguanidine (MIBG) scans. On CT (**a**, **b**), 2 avidly enhancing soft tissue masses (small and large red arrows) are identified in the left retroperitoneum with the larger lesion at the level of the IMA likely originating from the organ of Zuckerkandl. Both lesions are hyperintense on fat-suppressed T2-weighted axial images (**c**, **d**, small and large red arrows) and demonstrate increased uptake on a I-123 MIBG scan (**e**, small and large red arrows). Both were pathologically proven paragangliomas

**Fig. 11:**
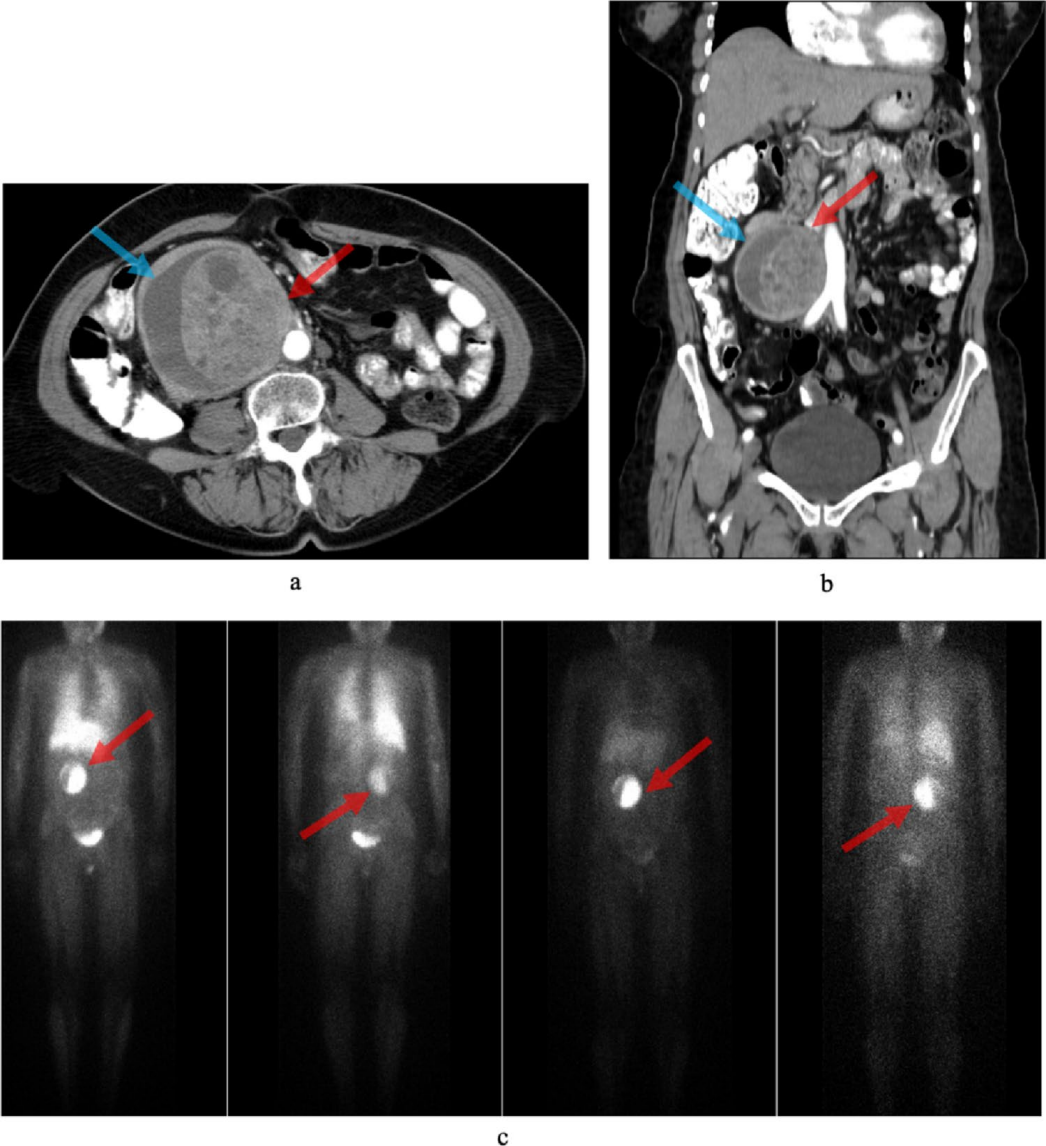
Axial (**a**) and coronal (**b**) contrast-enhanced CT scans through the abdomen demonstrate a large predominantly solid right retroperitoneal mass (red arrow) with a smaller crescentic cystic component (blue arrow) at the level of the organ of Zuckerkandl. A subsequent I-123 MIBG scan (**c**) shows radiotracer uptake within the larger solid component (red arrows). A diagnosis of paraganglioma was confirmed on surgical resection

Nuclear imaging with I-123 metaiodobenzylguanidine (MIBG) or Ga-68 DOTATATE are effective methods for confirming the diagnosis of paraganglioma. [8] (Figs. 10 and 11).

## Extrarenal angiomyolipomas

Angiomyolipomas (AMLs) are rare benign soft tissue tumors which contain smooth muscle, thickened blood vessels, and mature adipose tissue. [9] While AMLs are usually of renal origin, they can also present in other parts of the body, including the retroperitoneum. Extrarenal retroperitoneal AMLs are extremely rare. While the majority of cases are incidental [11], similar to renal AML, extrarenal retroperitoneal AML also have a propensity to hemorrhage and larger tumors are associated with a high bleeding risk, especially tumors larger than 4 cm in diameter. [9–10, 12] Consequently, some patients may present with abdominal pain and hemorrhagic shock, although most cases are identified incidentally. The majority of reported extrarenal retroperitoneal AMLs are benign, although a few rare cases of metastatic and recurrent AMLs have been reported in the literature. [10–11] Variants of extrarenal AMLs, such as the epithelioid variant, are considered to be more aggressive and may have higher rates of metastatic spread. [10–11]

**Fig. 12:**
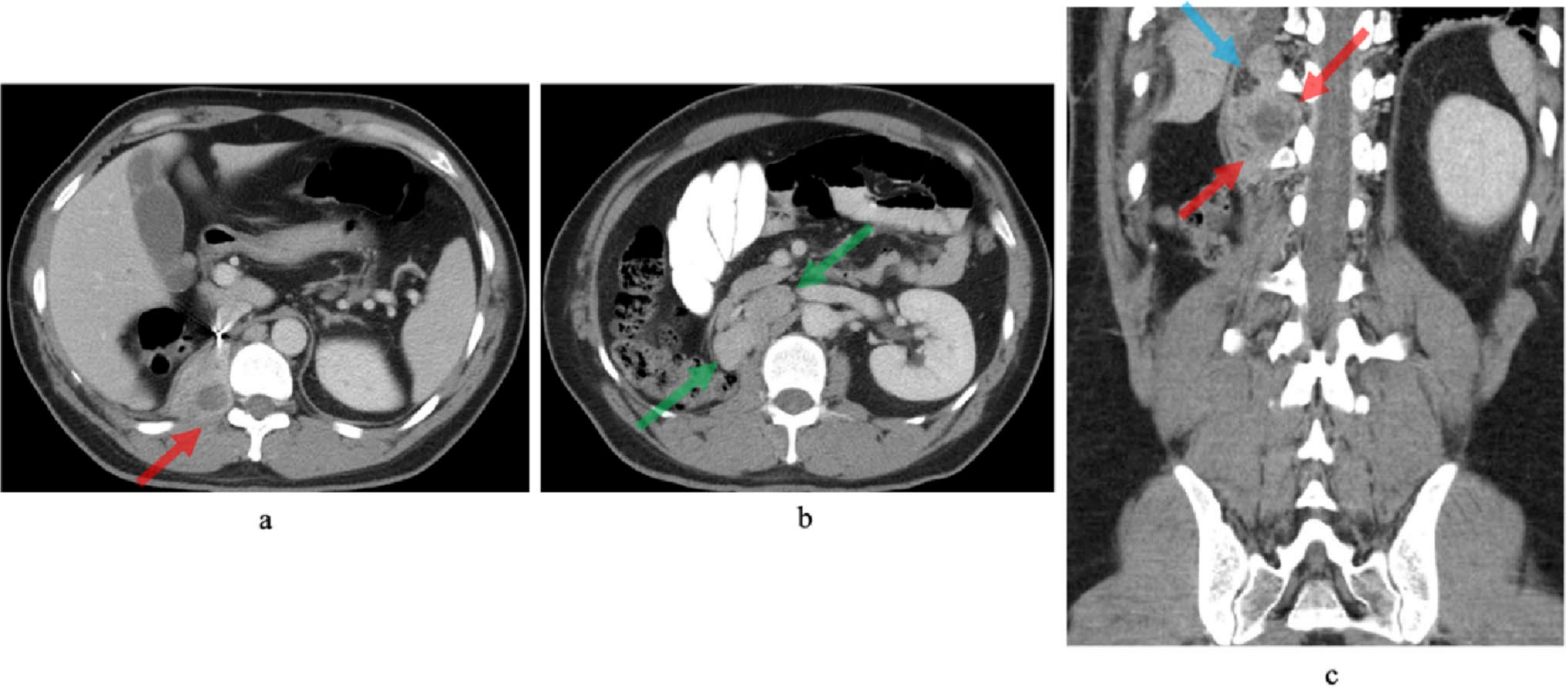
Retroperitoneal AML. Axial (**a**) and coronal (**c**) contrast-enhanced CT images show a heterogeneously enhancing right retrocrural mass (red arrows) with a small fat containing component (blue arrow). An axial (**b**) contrast-enhanced CT image through the mid-abdomen shows enhancing soft tissue masses in the retrocaval and aortocaval regions (green arrows). Surgical clips are from prior right nephrectomy

**Fig. 13:**
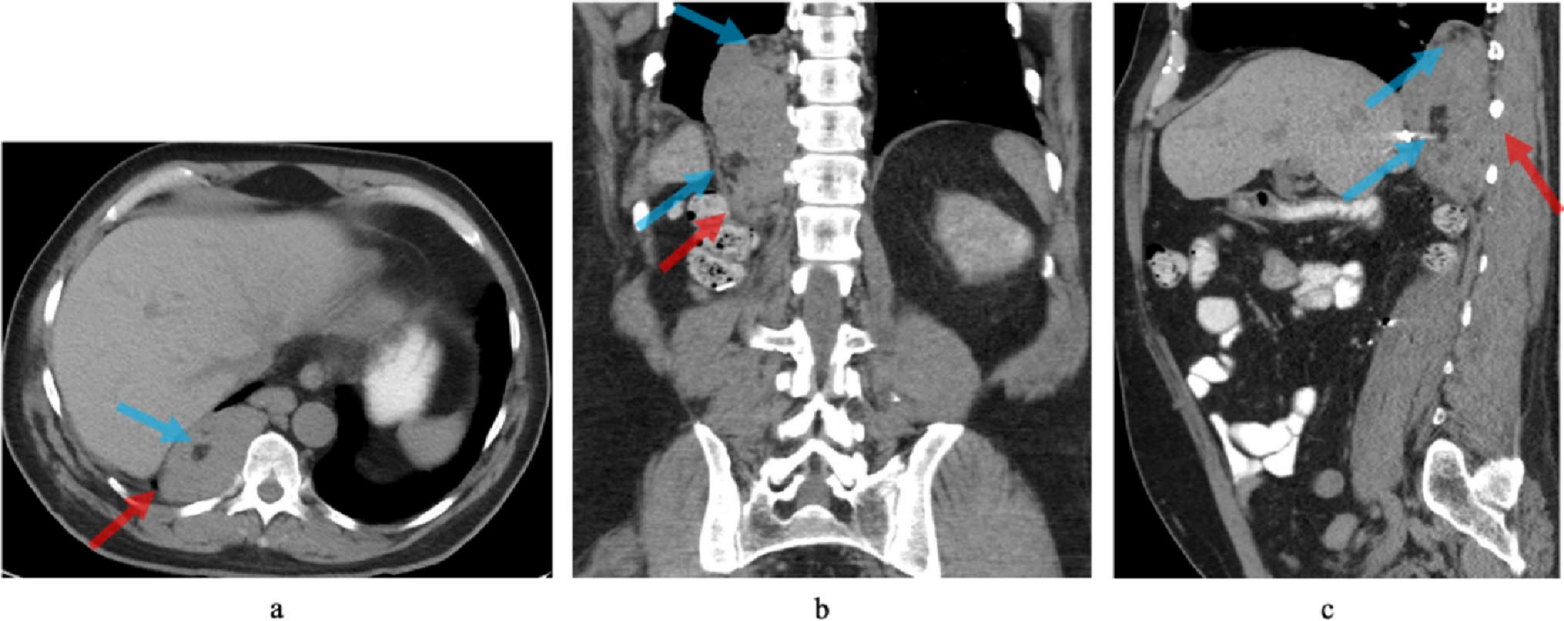
Subsequent axial (**a**), coronal (**b**) and sagittal (**c**) non-contrast CT images show a larger right retrocrural mass (red arrows) with fat containing components (blue arrows). The patient underwent surgical excision of the retroperitoneal enhancing masses and biopsy of the right retrocrural mass with a pathological diagnosis of angiomyolipoma

Imaging diagnosis of extrarenal retroperitoneal AML is challenging due to an overlap of imaging features with other malignant retroperitoneal entities such as liposarcoma. [9] Some extrarenal AMLs may not demonstrate fat content. [10] Often, the final diagnosis is heavily reliant on pathologic analysis. [9, 12] CT is the most common modality used in the diagnosis of retroperitoneal AMLs. CT findings of extrarenal AML overlap with renal AML, with most tumors containing areas of fat density. Studies of extrarenal AMLs have found several imaging features of AML that may help differentiate extrarenal AML from liposarcomas, particularly in the perinephric space. These include intratumoral linear vascularity, aneurysmal dilatation of intratumoral vessels, bridging veins, and hematoma. [13] (Figs. 12 and 13).

**Fig. 14:**
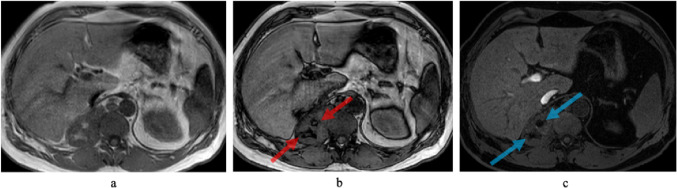
Axial opposed-phase (**a**, **b**) and fat-suppressed (**c**) images demonstrate ‘India ink’ chemical shift artifact on the out-of-phase image (red arrows) and loss of signal on the fat-suppressed image (blue arrows), findings consistent with intralesional fat

MRI can help delineate the extrarenal AML from surrounding anatomical structures as well as identify its vascular supply. [11] MRI findings include evidence of intralesional fat (such as ‘India ink’ chemical shift artifact along the periphery of the fat-containing portion, as well as loss of signal on fat-suppressed sequences compared to non-fat-suppressed sequences). Prominent vascularity may be seen. (Figure 14) [11–12].

## Castleman disease

Castleman disease is a relatively rare entity characterized by lymphocytic proliferation and involves the abdomen and pelvis in approximately 12-39% of cases. [3, 14] Castleman disease is divided into two clinical categories: localized/unicentric and diffuse-type/multicentric. Localized disease is the more common presentation, with a median age of onset in the 4th decade. [14] The localized subtype carries a better prognosis with adequate surgical resection, while diffuse-type disease more frequently requires systemic treatment and has a worse prognosis. [3] Histopathologic classifications of Castleman disease include hyaline vascular, plasma cell, human herpesvirus 8-associated, and multicentric Castleman disease not otherwise specified. [3, 14–15]

**Fig. 15:**
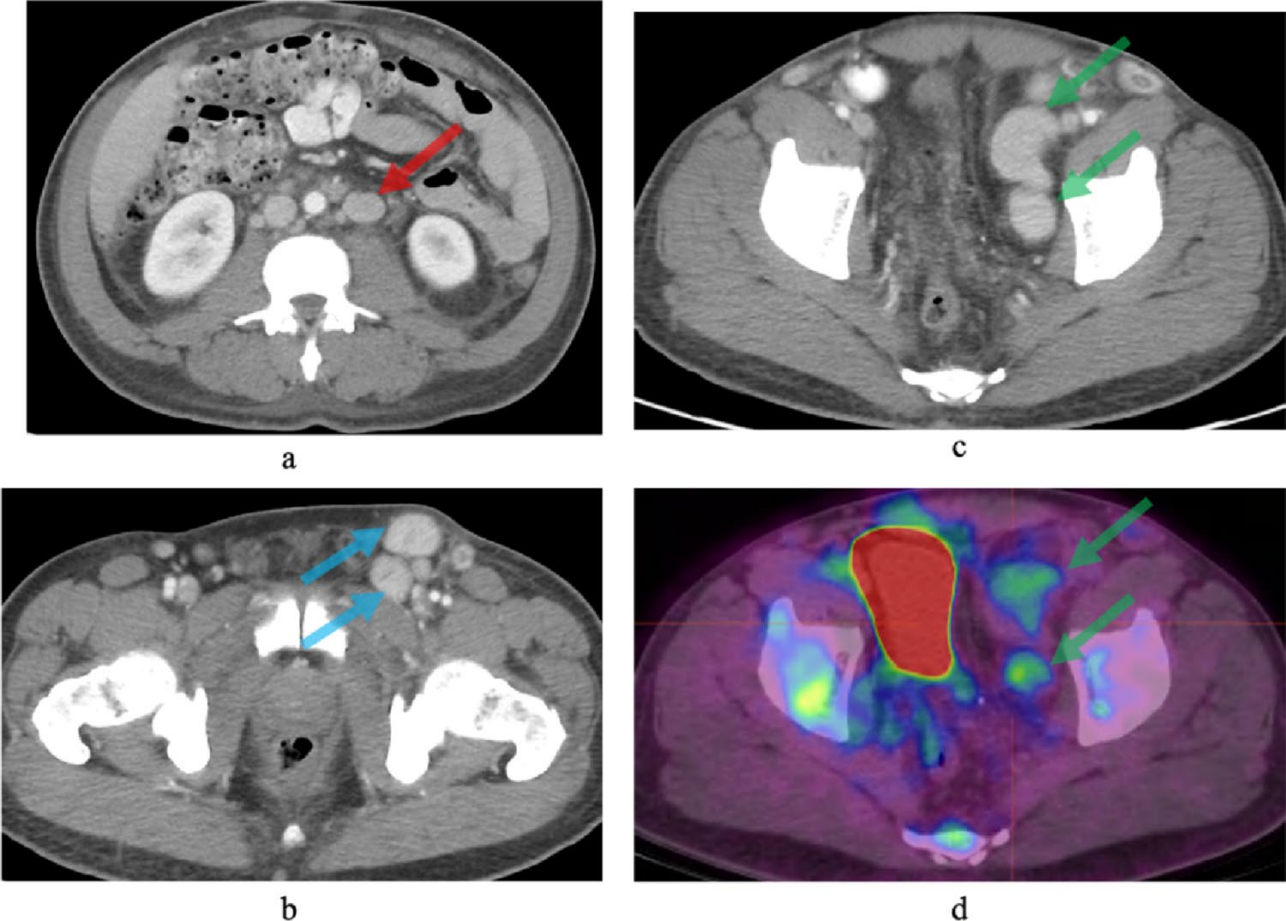
Castleman disease. Axial contrast-enhanced CT images through the retroperitoneum (**a**) and pelvis (**b**) show avidly enhancing lymphadenopathy in the left paraaortic (red arrow), left inguinal (blue arrow) and left external iliac (green arrow) chains. The left pelvic lymphadenopathy (green arrow) was FDG-avid on a subsequent PET scan (**d**). The patient had biopsy-proven Castleman’s disease

Diffuse-type or multicentric Castleman disease presents with multiple enlarged lymph nodes, which are usually well-circumscribed, homogeneous, and demonstrate mild to moderate enhancement on CT. [3] (Figure 15) Other findings may include hepatosplenomegaly, ascites, anasarca, and rarely, retroperitoneal fascial thickening. [3, 14] PET/CT can be useful for detecting the most suitable target for biopsy and for monitoring disease progression. Involved nodes or masses typically demonstrate only mild to moderate uptake with a mean maximum standardized uptake value (SUV_max_) of 5.6 to 5.8. [14]

Localized or unicentric Castleman disease tends to present on imaging with a large soft tissue mass on CT, which may or may not have adjacent satellite nodules. These soft tissue masses are usually well-circumscribed and large, and may be located in the mesentery, retroperitoneum, or both. [3, 14] Internal cystic change and necrosis can be seen, and rarely calcifications. Masses typically demonstrate strong homogeneous enhancement in the arterial phase, which persists on delayed phase image, especially when confined to the chest. However, lesions in the abdomen and pelvis are more likely to demonstrate heterogeneous enhancement. [14] On MRI, masses are typically hypointense or isointense to skeletal muscle on T1-weighted images and hyperintense on T2-weighted images. [3, 14] They typically demonstrate diffusion restriction. [12] Ultrasound may demonstrate well-defined, homogeneous, hypoechoic lesions with posterior acoustic enhancement. [14, 16] If no internal vascularity is seen, these masses can mimic cysts. [14]

When masses from localized Castleman disease present in the retroperitoneum, they can mimic retroperitoneal leiomyosarcoma. A helpful differentiating imaging finding in Castleman disease is an early and intense enhancement pattern. [3] In addition, Castleman disease tends to affect younger adults than those typically diagnosed with retroperitoneal leiomyosarcoma. [3]

## Retroperitoneal angiosarcomas

Angiosarcoma is a rare and aggressive soft tissue sarcoma which arises from vascular endothelial cells. [17] While these tumors can occur at any age, they are most common in adults aged 60-70 years. [17] While angiosarcomas occur most frequently in the skin and superficial soft tissues, they can also occur in the retroperitoneum and visceral organs, with approximately 10% of reported cases occurring in the retroperitoneum. [17–18] Risk factors for the development of angiosarcoma include prior radiation therapy, lymphedema, and chemical exposure (e.g. vinyl chloride). [17, 19] There are also several familial syndromes and conditions associated with angiosarcoma, such as Neurofibromatosis type 1, Maffucci syndrome, Klippel-Trenaunay syndrome, hemochromatosis, and bilateral retinoblastomas. [17]

Clinically, retroperitoneal angiosarcomas are more likely to present with intratumoral hemorrhage and associated pain, compared to most other retroperitoneal tumors. [18] Patients with retroperitoneal angiosarcoma may also present with symptoms related to mass effect or invasion of these tumors into surrounding organs. [17] Resultant hematomas can sometimes obscure the diagnosis of these tumors. [18] Angiosarcomas spread hematogenously, most commonly to the lungs. Other common sites of metastatic involvement include the liver, bones, soft tissues, and lymph nodes. [17]

**Fig. 16:**
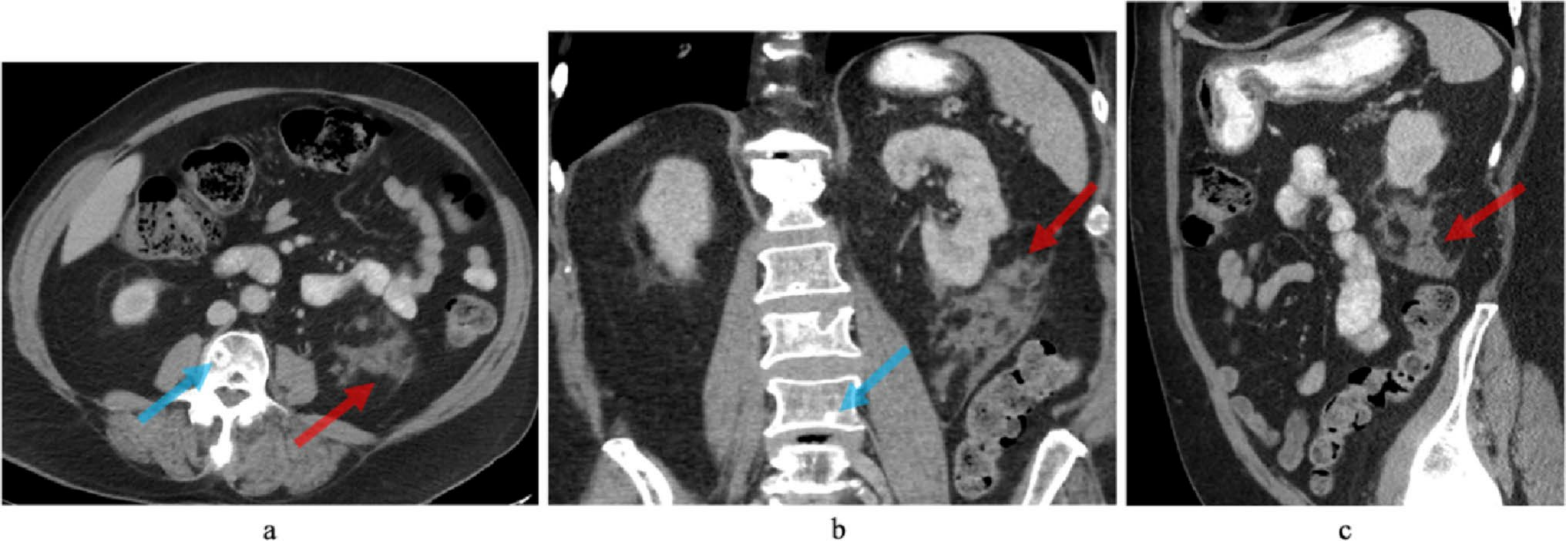
Retroperitoneal Angiosarcoma. Axial (**a**), coronal (**b**) and sagittal (**c**) contrast-enhanced CT images demonstrate multiple ill-defined enhancing soft tissue nodules in the left inferior perinephric space (red arrows) due to biopsy-proven angiosarcoma. The patient also had a history of prostate cancer with bony metastatic disease (blue arrow)

On CT, retroperitoneal angiosarcomas may present as an enhancing soft tissue mass with irregular margins. [17] (Figure 16) Soft tissue calcifications may be seen. If advanced disease is present, there may be invasion of adjacent organs or bone. [17] MR imaging of angiosarcomas typically demonstrates intermediate signal on T1-weighted images and hyperintense signal on T2-weighted images. Foci of high T1 signal may be seen in areas of adjacent hemorrhage. [17] Tumors typically enhance after contrast administration, with possible areas of central necrosis. Intratumoral vessels may exhibit characteristics of high flow or low flow. [17] On diffusion-weighted imaging, angiosarcomas typically have very low apparent diffusion coefficient. [17]

Ultrasound findings of retroperitoneal angiosarcoma are nonspecific and not well reported. One case report of primary retroperitoneal angiosarcoma reported an irregular, heterogeneously hypoechoic lesion in the retroperitoneum without abundant internal Doppler flow. [19] Ultimately, cross-sectional imaging with CT, MRI, or PET-CT is required to fully delineate the tumor and its surrounding anatomy. [19]

## Retroperitoneal hemangiomas and cystic lymphangiomas

Hemangiomas are benign tumors which typically involve the soft tissues, but can be rarely seen in the retroperitoneum. Cystic lymphangiomas are relatively more common in the retroperitoneum compared to hemangiomas, although both are exceedingly rare. [18, 20]

Hemangiomas are benign vascular tumors. In adults, retroperitoneal hemangiomas comprise approximately 1-3% of all reported retroperitoneal masses. [21] They generally arise from retroperitoneal organs, such as the kidney, adrenal glands, or pancreas. No apparent gender predilection has been found. [20–21] In most cases, the lesion is detected incidentally. [20] Although retroperitoneal hemangiomas are benign, they can compress or invade local structures. Some patients have presented with flank pain, hematuria, renal vein thrombosis, or hydronephrosis. [20–21] Retroperitoneal hemangiomas have the potential to rupture and may also present as massive hemorrhage, which may necessitate surgical intervention. [20] When retroperitoneal hemangiomas grow large in size, they may also exhibit other complications such as thrombosis and obstruction of adjacent venous structures. [20]

**Fig. 17:**
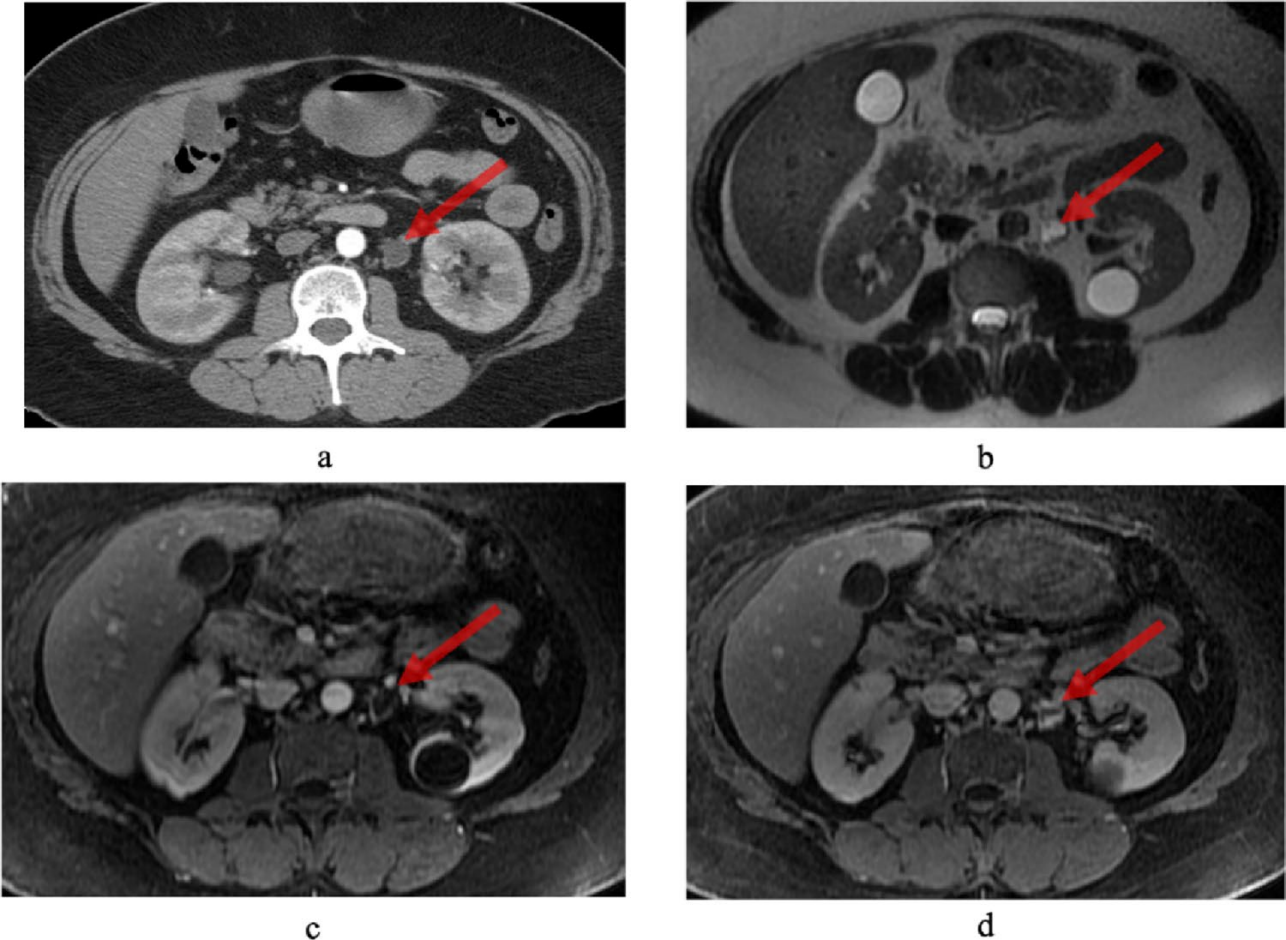
Retroperitoneal Hemangioma. An axial contrast-enhanced CT image (**a**) shows a hypodense left paraaortic lesion with incomplete peripheral enhancement (red arrow). The lesion is hyperintense on an axial T2-weighted image (**b**) and demonstrates incomplete peripheral nodular enhancement with progressive centripetal fill-in on axial fat suppressed contrast-enhanced T1-weighted images (**c**, **d**), features compatible with a retroperitoneal hemangioma

Imaging features of these lesions are nonspecific. On CT, retroperitoneal hemangiomas may present as a heterogeneously enhancing lesion with a dense or calcified peripheral rim. [20, 22–23] Other imaging characteristics have also been described, including a cystic mass with mild enhancement and a homogeneously hypodense and hypoenhancing mass. [21] (Figure 17) MRI may demonstrate internal hypointense signal on T1-weighted images, as well as hyperintense signal on T2-weighted images, which is thought to represent areas of hemorrhage and tissue hyalinization. [20–21] Ultrasound features of retroperitoneal hemangiomas are also non-specific and can range from hyperechoic to anechoic. Posterior acoustic enhancement may be seen. [23]

Lymphangiomas are benign tumors of the lymphatic system, with three histological subtypes: cystic, capillary, and cavernous. Retroperitoneal lymphangiomas comprise less than 1% of all lymphangiomas and are most commonly of the cystic type. [24] They are usually asymptomatic, but may present with symptoms such as abdominal discomfort or abdominal distention if the mass grows to a large size and surrounds or compresses adjacent organs. [23] Rarely, back pain, anemia, ureteric obstruction, and hematuria have also been reported. Superinfection of cystic lymphangiomas can also occur. [25]

**Fig. 18:**
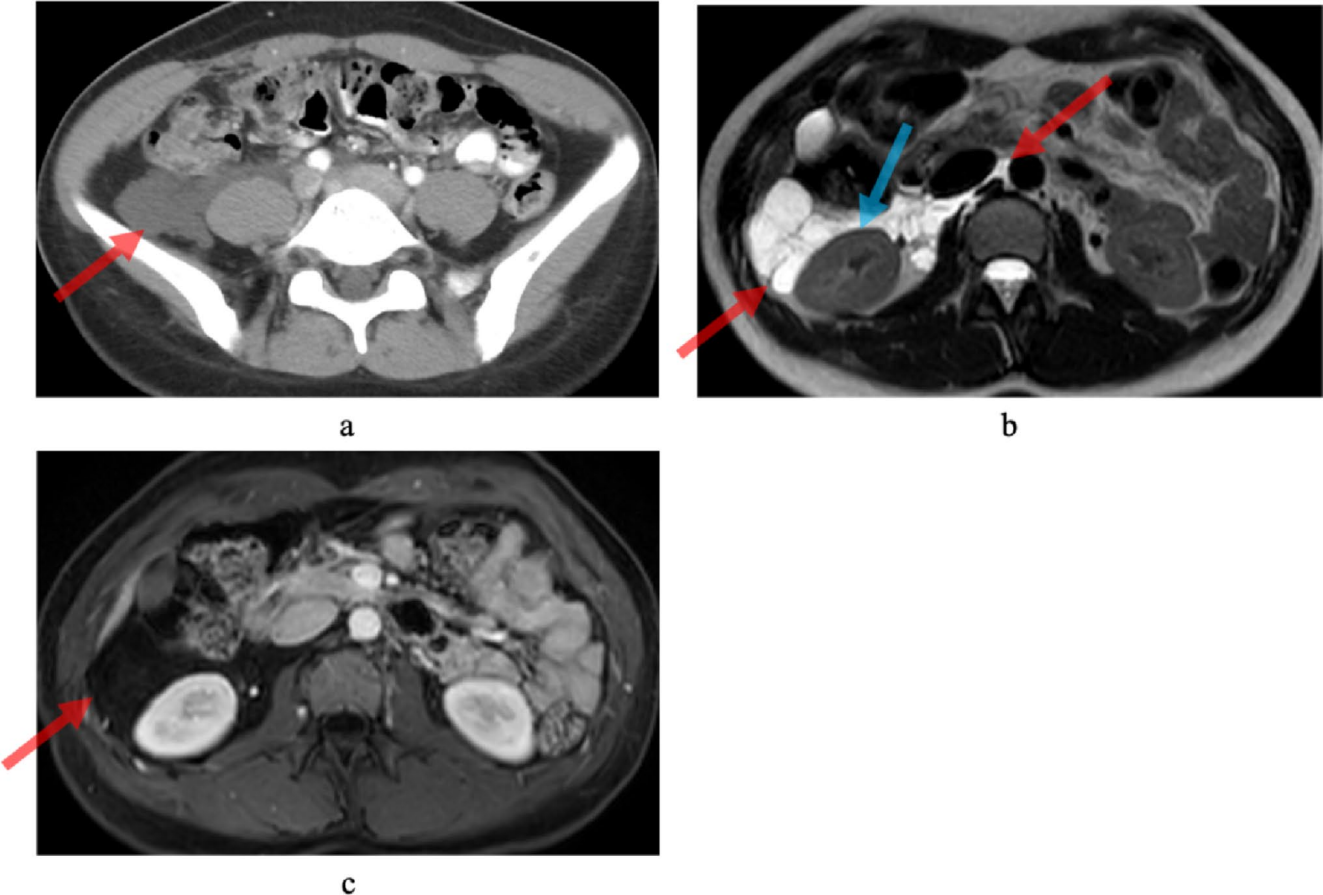
Retroperitoneal Lymphangioma. An axial contrast-enhanced CT image (**a**) shows a lobulated low-density mass within the right inferior retroperitoneum (red arrows). An axial T2-weighted image (**b**) shows a multi-septated cystic right retroperitoneal mass (red arrows) insinuating around the right kidney (blue arrow). The mass is non-enhancing on an axial fat-saturated T1-weighted contrast enhanced image (**c**)

Cystic lymphangiomas most commonly present on imaging as uniseptated or multiseptated cystic masses, with fluid or fat density within the cystic components. [26] Calcifications are rare. [24] On CT, retroperitoneal lymphangiomas typically present as well-circumscribed homogeneous cystic lesions with septations. On ultrasound, a fluid-containing lesion with loculations can be seen in macrocystic lesions. Microcystic lymphangiomas can have a more nonspecific hyperechoic appearance on US due to multiple tissue interfaces between small cystic foci. [27] MRI typically demonstrates internal low T1 signal and high T2 signal. (Figure 18). Superinfected lymphangiomas may present with heterogeneous T2-weighted signal with fluid-fluid levels and delayed enhancement of the external walls and septa, and may demonstrate internal heterogeneity on CT and ultrasound. Internal hemorrhage can also occur, leading to high T1 signal and low T2 signal. [25–26] Chylous material may also lead to low or negative CT attenuation values or signal drop-out on out-of-phase MR sequences. [25]

## Retroperitoneal inflammatory myofibroblastic tumor

Inflammatory myofibroblastic tumor (IMT) is a rare tumor of unknown etiology. [28–29] It was previously described as part of the spectrum of ‘inflammatory pseudotumors’ but more recently has been defined as a separate entity. [28–29] While IMTs tend to have a benign course, they are classified as low-grade malignancies. Histologic diagnosis is characterized by the presence of proliferative myofibroblasts or fibroblasts with infiltration of inflammatory cells, such as lymphocytes, histiocytes, and plasma cells. [29] The most common site of involvement is the lung. Retroperitoneal involvement is rare. [29–30] Patients can present with fever and weight loss. [30]

**Fig. 19:**
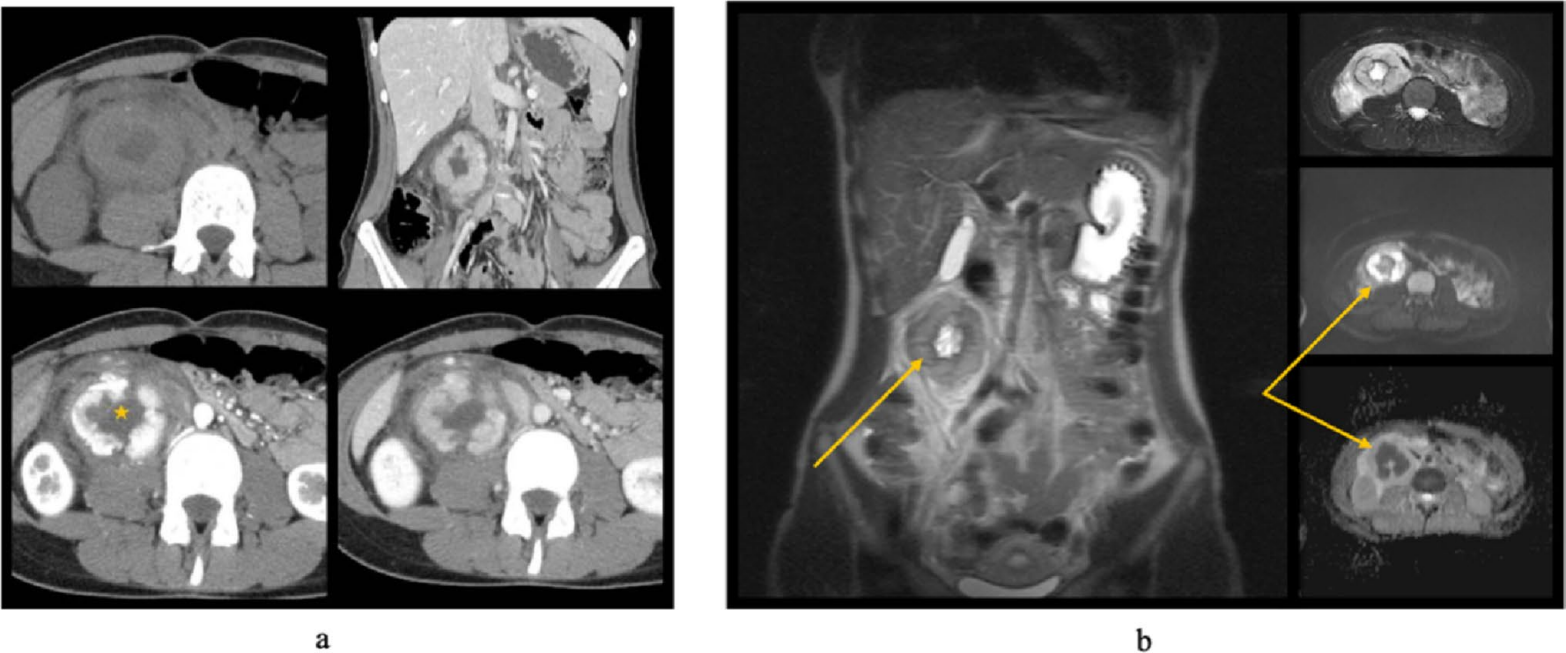
Retroperitoneal Inflammatory Myofibroblastic Tumor. Axial non-contrast, axial contrast-enhanced arterial and portal venous phase and coronal contrast-enhanced CT images (**a**) show a hypervascular mass with a hypodense necrotic center (orange asterisk) displacing the right kidney. MRI including coronal T2-weighted, axial fat-saturated T2-weighted, diffusion weighted (b100) and apparent diffusion coefficient images (**b**) show high restriction diffusion in the solid components of the mass (orange arrows). Reproduced with permission: [34]

Imaging features of retroperitoneal IMTs have not been well established. IMTs in other locations have been described, including heterogeneous or homogeneous masses, with or without central necrosis, with variable enhancement patterns on CT and MRI. [29] Imaging features may depend on the inflammatory and fibrous composition of the tumor. [31] IMTs may be well-circumscribed or infiltrating, although retroperitoneal IMTs are more likely to be well-circumscribed. [31] Lesions tend to be persistently enhancing and may demonstrate heterogeneous enhancement if internal cystic foci are present. [32] On MRI, retroperitoneal IMTs tend to demonstrate isointense to slightly hyperintense signal on T1-weighted images, hyperintense signal on T2-weighted images, and variable enhancement. [31] Ultrasound has also demonstrated a varied appearance, including variable echogenicity and prominent Doppler vascular flow. [29, 31] Hypermetabolism has been reported in retroperitoneal IMTs on F18-FDG PET-CT, which may mimic other malignancies such as lymphoma. [33] (Figure 19).

## Conclusion

Vascular retroperitoneal tumors are rare and may be benign or malignant. These tumors often present with vague symptoms, making diagnosis challenging. Imaging findings may also be non-specific. As a result, knowledge of these entities can be helpful in suggesting a differential diagnosis. Cross-sectional imaging with CT and MRI is valuable to help characterize the mass and delineate its relationship with the surrounding anatomy and aid in potential surgical planning.

## Data Availability

No datasets were generated or analysed during the current study.

## References

[1] Sangster GP, Migliaro M, Heldmann MG, et al. The gamut of primary retroperitoneal masses: multimodality evaluation with pathologic correlation. *Abdominal Radiology*. 2016; 41: 1411–1430. 27271217 10.1007/s00261-016-0735-6

[2] Hartmann DS, Hayes WS, Choyke PL, Tibbetts GP. Leiomyosarcoma of the Retroperitoneum and Inferior Vena Cava: Radiologic-Pathologic Correlation. *RadioGraphics*. 1992; 12: 1203–1220. 1439022 10.1148/radiographics.12.6.1439022

[3] Marko J, Wolfman DJ. Retroperitoneal Leiomyosarcoma. *RadioGraphics*. 2018; 38: 1403–1420. 30207936 10.1148/rg.2018180006 PMC6166742

[4] Wang MX, Menias CO, Elsherif SB, Segaran N, Ganeshan D. Current update on IVC leiomyosarcoma. *Abdominal Radiology*. 2021; 46: 5284–5296. 34415408 10.1007/s00261-021-03256-9

[5] Webb EM, Wang ZJ, Westphalen AC, Nakakura EK, Coakley FV, Yeh BM. Can CT Features Differentiate Between Inferior Vena Cava Leiomyosarcomas and Primary Retroperitoneal Masses? *AJR*. 2013; 200: 205–209. 23255763 10.2214/AJR.11.7476

[6] Gage MJ, Patel AV, Koenig KL, Newman E. Non-Vena Cava Venous Leiomyosarcomas: A Review of the Literature. *Ann Surg Oncol*. 2012. 19: 3368–3374. 22618717 10.1245/s10434-012-2379-2

[7] Matsuzono T, Chan CY, Chan MY. Gonadal vein leiomyosarcoma: A case report with radiological findings. *Intractable & Rare Diseases Research*. 2015; 4(3): 152–154. 26361567 10.5582/irdr.2015.01016 PMC4561245

[8] Carrasquilllo JA, Chen CC, Jha A, et al. Imaging of Pheochromocytoma and Paraganglioma. *J Nucl Med*. 2021; 62: 1033–1042. 34330739 10.2967/jnumed.120.259689 PMC8833868

[9] Tseng CA, Pan YS, Su YC, Wu DC, Jan CM, Wang WM. Extrarenal retroperitoneal angiomyolipoma: case report and review of the literature. *Abdominal Imaging*. 2004; 29: 721–723. 15185030 10.1007/s00261-004-0179-2

[10] Gupta C, Malani AK, Gupta V, Singh J, Ammar H. Metastatic retroperitoneal epithelioid angiomyolipoma. *J Clin Pathol*. 2007; 60: 428–431. 17405979 10.1136/jcp.2006.039503 PMC2001107

[11] MinjaEJ, Pellerin M, Saviano N, Chamberlain RS. Retroperitoneal extrarenal angiomyolipomas: evidence-based approach to a rare clinical entity. *Case Rep Neph*. 2012; 2012: 374107. 10.1155/2012/374107 10.1155/2012/374107 PMC3914176 24555133

[12] Wroclawski M, Baccaglini W, Pazeto CL, et al. Extrarenal Angiomyolipoma: differential diagnosis of retroperitoneal masses. *Int Braz J Urol*. 2018; 44: 639–41. 29211401 10.1590/S1677-5538.IBJU.2016.0670 PMC5996806

[13] Wang L, Wong Y, Chen C, See L. Computerized tomography characteristics that differentiate angiomyolipomas from liposarcomas in the perinephric space. *J Urol*. 2002; 167: 490–493. 11792904 10.1016/S0022-5347(01)69071-2

[14] Pitot MA, Tahboub Amawi AD, Alexander LF, et al. Imaging of Castleman Disease. *RadioGraphics*. 2023; 43(3): e220210. 37471247 10.1148/rg.220210

[15] Bonekamp D, Horton KM, Ralph RH, Fishman EK. Castleman Disease: The Great Mimic. *RadioGraphics*. 2011; 31: 1793–1807. 21997995 10.1148/rg.316115502

[16] Zhou W, Zhan W, Zhou J, Zhu Y, Yao J. Sonographic Findings of Localized Castleman Disease of the Abdomen and Pelvis. *J Clin Ultrasound*. 2014; 43: 401–405. 25346169 10.1002/jcu.22245

[17] Gabellah AH, Jensen CT, Palmquist S, et al. Angiosarcoma: clinical and imaging features from head to toe. *Br J Radiol*. 2017; 90: 20170039. 28471264 10.1259/bjr.20170039 PMC5594986

[18] Improta L, Tzanis D, Bouhadiba T, Abdelhafidh K, Bonvalot S. Overview of primary adult retroperitoneal tumours. *Eur J Surg Onc*. 2020; 46: 1573–1579. 10.1016/j.ejso.2020.04.054 32600897

[19] Chen BQ, Luo WW, Lin WJ, Zhuang W, Li SL. Primary retroperitoneal angiosarcoma: A case report. *Open Life Sci.* 2023; 18: 20220546. 36874627 10.1515/biol-2022-0546 PMC9975949

[20] Mossanen M, Dighe M, Gore J, Mann G. Large retroperitoneal hemangioma encompassing the renal vein. *Can Urol Assoc J.* 2015; 9(11-12): E894-6. 26834900 10.5489/cuaj.3356 PMC4707912

[21] Debaibi M, Sghair A, Sahnoun M, et al. Primary retroperitoneal cavernous hemangioma: An exceptional disease in adulthood. *Clin Case Rep*. 2022; 10: e05850. 35592049 10.1002/ccr3.5850 PMC9097370

[22] Khazaal AS, Sultan OM, Rasheed IAAM. Radio-pathological diagnosis of a retroperitoneal cavernous hemangioma. *J Surg Case Rep*. 2023; 3: 1–3. 10.1093/jscr/rjad095 PMC9991573 36896163

[23] Godar M, Yuan Q, Shakya R, Xia Y, Zhang P. Mixed Capillary venous retroperitoneal hemangioma. *Case Rep Radiol*. 2013; 2013:258352. 10.1155/2013/258352 PMC3600266 23533905

[24] Mansour S. Kluger Y, Khuri S. Adult Primary Retroperitoneal Lymphangioma: Updated Facts. *World J Oncol*. 2023; 14(1): 15–20. 36896002 10.14740/wjon1561 PMC9990737

[25] Raufaste Tistet M, Ernst O, Lanchou M, Vermersch M, Lebert P. Imaging features, complications and differential diagnoses of abdominal cystic lymphangiomas. *Abdominal Radiology*. 2020; 45: 3589–3607. 32296900 10.1007/s00261-020-02525-3

[26] Yang DM, Jung DH, Kim H, et al. Retroperitoneal Cystic Masses: CT, Clinical, and pathologic findings and literature review. *RadioGraphics*. 2004; 24(5) 10.1148/rg.245045017 15371613

[27] Hoang VT, Nguyen MD, Van HAT, Hoang DT. Review of diagnosis, differential diagnosis, and management of retroperitoneal lymphangioma. *Japanese Journal of Radiology*. 2023. 41: 283–301. 36327088 10.1007/s11604-022-01356-0

[28] Attili SVS, Chandra CR, Hemant DK, Bapsy PP, RamaRao C, Anupama G. Retroperitoneal inflammatory myofibroblastic tumor. *World J Surg Onc*. 2005; 3: 66. 10.1186/1477-7819-3-66 PMC1276822 16212671

[29] Lee SB, Yoon JH, Kim SH, Lee Y, Lee JS, Seo JW. A Retroperitoneal Inflammatory Myofibroblastic Tumor Mimicking a Germ Cell Tumor of the Undescended Testis: A Case Report and Literature Review. *Advances in Computed Tomography*. 2016; 5: 35–41.

[30] Koirala R, Shakya V, Agrawal CS, et al. Retroperitoneal inflammatory myofibroblastic tumor. *Am J Surg*. 2010; 199: e17-e19. 19837393 10.1016/j.amjsurg.2009.04.014

[31] Surabhi VR, Chua S, Patel RP, Takahashi N, Lalwani N, Prasad SR. Inflammatory Myofibroblastic Tumors. *Radiol Clin N Am*. 2016; 54: 553–563. 27153788 10.1016/j.rcl.2015.12.005

[32] Tan HN, Wang B, Xiao HJ, Lian YB, Gao JB. Radiologic and Clinicopathologic Findings of Inflammatory Myofibroblastic Tumor. *J Comput Assist Tomogr*. 2017; 41: 90–91. doi: 10.1097/RCT.0000000000000444 27224222 10.1097/RCT.0000000000000444

[33] Wakankar R. An Interesting Case of Inflammatory Myofibroblastic Tumor Masquerading as Lymphoma Detected on 18F-FDG PET-CT. *Cureus*. 2023; 15(9): e44652. doi: 10.7759/cureus.44652 37799228 10.7759/cureus.44652 PMC10549778

[34] Carlos O, Rui C, Amélia E, Filipe CA. Retroperitoneal inflammatory myofibroblastic tumor: a case report. Eur J Radiol Open. 2017;4:9–12. 10.1016/j.ejro.2017.01.003 . PMID: 28275656; PMCID: PMC5328934 28275656 10.1016/j.ejro.2017.01.003 PMC5328934

